# An exploratory study of the effects of strenuous exercise on markers of coagulation activation, circulating microparticles, and inflammation in sickle cell trait

**DOI:** 10.1002/jha2.23

**Published:** 2020-05-26

**Authors:** Sarah Skinner, Eric D. Ryan, Harry C. Stafford, Robert G. McMurray, Nigel S. Key, Micah J. Mooberry

**Affiliations:** ^1^ Hematology/Oncology Division and UNC Blood Research Center University of North Carolina at Chapel Hill Chapel Hill North Carolina USA; ^2^ Department of Exercise and Sport Science University of North Carolina at Chapel Hill Chapel Hill North Carolina USA; ^3^ Departments of Family Medicine and Orthopaedics University of North Carolina at Chapel Hill Chapel Hill North Carolina USA

## Abstract

This exploratory study evaluated the effect of intense exercise on biomarkers of inflammation and coagulation activation in subjects with and without sickle cell trait (SCT). Fifteen healthy African American men (18‐35 years, 5 SCT, 10 control) completed a strenuous exercise protocol. Microparticle‐associated prothrombinase and tissue factor activities, as well as soluble VCAM, total white cell and monocyte count increased transiently in all subjects following exercise. In the SCT group, exercise resulted in increased d‐dimer, erythrocyte phosphatidylserine exposure, as well as increased circulating erythrocyte‐ and endothelial‐derived microparticle numbers. These alterations could contribute to exercise‐related complications in people with SCT.

## 1 INTRODUCTION

It is well established that regular exercise training lowers cardiovascular, metabolic, and thromboembolic disease risk [1, 2]. Paradoxically, strenuous acute exercise may transiently increase the risk of cardiovascular and thromboembolic complications, and is associated with a transient hypercoaguable state and elevated markers of inflammation [3]. Microparticles (MPs), membrane‐derived vesicles (0.1‐1 µm) that are released from cells during apoptosis or upon activation, have been shown to contribute to coagulation activation following acute exercise [4]. The prothrombotic properties of MPs may be related to several mechanisms, including the exposure of phosphatidylserine (PS) or tissue factor (TF) on the outer membrane leaflet [5]. PS exposure provides a docking surface for coagulation enzymatic complexes, and provides a catalytic surface for assembly of the tenase and prothrombinase complexes, thereby promoting thrombin generation. TF is an integral‐membrane protein that triggers the extrinsic pathway of coagulation, when bound to activated factor VII.

Accumulating evidence suggests that sickle cell trait (SCT), the heterozygous form of sickle cell anemia (SCA), could be a risk factor for exercise‐related complications, including rhabdomyolysis or sudden death [6]. Although SCT has generally been considered to be benign, previous studies have shown that hemorheological and inflammatory responses to exercise may be altered in individuals with SCT [7]. However, the pathogenesis of exercise‐related complications in SCT has not been explored, and the effect of exercise on red blood cell (RBC) physiology and circulating MP concentrations in this population remains unknown.

The present study set out to explore short‐term effects of a single bout of vigorous exercise on a variety of biomarkers of activation of coagulation and inflammation in subjects with and without SCT.

## 2 METHODS

The study included 15 healthy African American males (18‐35 years). Ten of the subjects had normal adult hemoglobin (controls), while 5 had SCT. During a primary visit, subjects completed a graded exercise test to determine maximal oxygen consumption (VO_2_max). Then, during a second visit, subjects completed a single bout of exercise, composed of 30 min of treadmill running at 65% of their VO_2_max, followed by an increase in treadmill grade by 2.5% every 3 min until volitional exhaustion. Blood was collected at baseline, immediately post‐exercise, and at 1 and 2 h post‐exercise. Analyses included complete blood count (CBC), RBC PS exposure (by flow cytometry), and plasma levels of d‐dimer, sVCAM, free hemoglobin (PF‐Hb), MP prothrombinase activity (Zymuphen MP‐activity assay), and MPTF activity, quantified using an in‐house chromogenic assay [8]. Flow cytometry was used to characterize MPs in platelet poor plasma collected at baseline, immediately after, and 2 h after exercise, by number and phenotype using the standardized ISTH protocol [9]. MPs were characterized as platelet MP (PMP:AnnV+/CD41+), endothelial MP (EMP:CD31+/CD41−), red cell MP (RBCMP:AnnV+/CD235a+), leukocyte MP (LMP:AnnV+/CD45+), or monocyte MP (MMP:AnnV+/CD14+).

Statistical analyses were carried out using Prism 8 (GraphPad San Diego, CA). Mann‐Whitney *U* tests were used to compare differences in age, body mass index (BMI), and VO_2_max between groups. Two‐way ANOVA tests were used to compare the effects of the exercise bout in the two groups. Tukey post hoc tests were used to evaluate the differences between different time points in all subjects. Sidak multiple comparisons tests were performed to determine between and within group differences at each time point. Significance was defined as *P* < .05.

## 3 RESULTS

**FIGURE 1:**
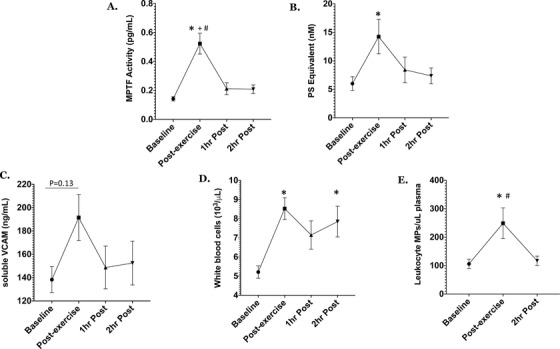
Changes in microparticle‐tissue factor (MPTF) activity (A), microparticle (MP) prothrombinase activity (PS equivalent) (B), soluble vascular adhesion molecule concentration (VCAM) (C), white blood cell concentration (D), and leukocyte MPs (E) after strenuous exercise in all subjects. Data are presented as mean ± SEM. ^*^ *P* < .05 compared to baseline; ^+^ *P* < .05 compared to 1 h post‐exercise; ^#^ *P* < .05 compared to 2 h post‐exercise

Subjects with SCT were similar to controls with respect to age, BMI, or VO_2_max. The results of the two‐way ANOVA tests showed that there was a significant effect of exercise on MP prothrombinase activity (*P* = .0003), MPTF activity (*P* = .028), sVCAM (*P* = .001), WBC (*P* < .0001), and LMPs (*P* = .009) in all subjects (n=15; Figure 1). Post‐hoc analyses revealed that there were transient increases in MP prothrombinase activity (*P* < .05) and MPTF activity (*P* < .001) in all subjects immediately post‐exercise. We also observed significant elevations in total white blood cell (WBC) and monocyte counts immediately after exercise in all subjects (*P* < .05), as well as a trend toward increased sVCAM (*P* = .13). LMPs increased significantly in all subjects immediately post‐exercise, and returned to baseline levels by 2 h post exercise (Figure 1). There were no significant interactions and no main group effects observed for these measures. No other significant differences in circulating MPs, PF‐Hb, or CBC were observed at any time point.

**FIGURE 2:**
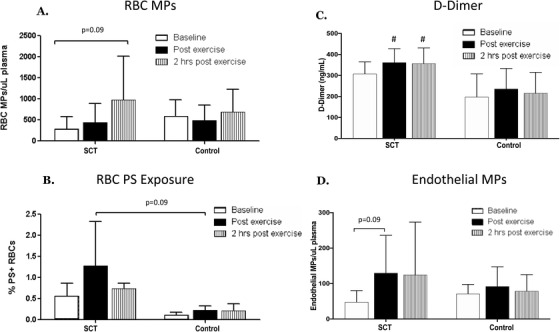
Changes in red blood cell microparticles (RBC MPs) (A), RBC PS exposure (%PS+RBCs) (B), d‐dimer (C), and endothelial cell MPs (endothelial MPs) (D) after strenuous exercise in the sickle cell trait (SCT) group compared to the control group. Data are presented as mean ± SEM. ^#^ *P* < .05 compared to control at the same time point

Several differing responses were observed in the subjects with SCT compared to controls. Our analyses revealed a significant effect (*P* = .01) for elevated RBC PS exposure in the SCT group. The post‐hoc analyses showed a trend toward increased RBC PS exposure immediately post‐exercise in SCT subjects compared to controls, (post‐exercise %PS+RBCs: SCT 1.28+/−1.05% vs control 0.22 +/−0.10%; *P* = .09, n = 4), as well as a trend toward increased RBCMPs in SCT subjects at 2 h post‐exercise (SCT: 291+/−284 RBCMPs/µL at baseline; 975+/−1033 RBC MPs/µL at 2 h; *P* = .13; Figure 2). EMP numbers also tended to increase post‐exercise in SCT subjects only (SCT: *P* = .09; Control: *P* = 0.38; Figure 2). Our results also showed a significant group effect (*P* = .02) and an effect of exercise (*P* = .007) on d‐dimer, however there was no significant interaction between these parameters. The post hoc analyses showed a trend toward elevated levels of d‐dimer in the SCT group compared to the control group at baseline (*P* = .11), which reached statistical significance post‐exercise (*P* = .05), and remained significantly elevated 2 h post‐exercise (*P* < .05) (Figure 2). No significant differences in PMP, LMP, or MMP numbers were observed between groups or time points.

## 4 DISCUSSION

This study demonstrates that an acute bout of strenuous exercise results in a transient increase in MPTF procoagulant activity accompanied by increased MP‐prothrombinase activity, WBC and monocyte counts, leucocyte MP counts, and sVCAM. Therefore, our results are similar to previous findings that strenuous exercise causes acute systemic activation of coagulation and inflammation [3], while providing insight into an additional mechanism through which rigorous exercise could increase thrombin generation. Elevated MPTF procoagulant activity has been observed in a number of prothrombotic and inflammatory disorders, indicating a potentially important role in the pathogenesis of thrombosis [10]. Although the cellular source of MPTF seen in this study is unknown, previous research has shown that the majority of TF‐expressing MPs are leucocyte‐derived, and that leukocytes are prone to microvesiculation when they are exposed to inflammatory cytokines [5]. In our study, both sVCAM and LMPs increased significantly post‐exercise. Therefore, it is possible that exercise‐related inflammation stimulated LMP formation, which contributed to increased MPTF activity. Future studies will need to be carried out to confirm this hypothesis.

The findings of this study also show that exercise resulted in trends for increased RBC PS exposure and elevated levels of circulating RBCMPs and EMPs in individuals with SCT. Elevated PS exposure could potentially increase the risk of exercise‐related complications in two ways. First, PS exposure increases erythrocyte adhesion, which can impede blood flow in the microcirculation [11]. Second, PS exposure activates coagulation by providing a surface for prothrombinase and tenase to assemble [10]. The elevated RBCMPs and EMPs could also potentially play a role in coagulation activation through TF‐dependent and independent mechanisms, and thereby increase the risk of developing exercise‐related complications [8].

Both RBC PS exposure and RBCMP formation occur as a result of eryptosis, or “suicidal” erythrocyte death, which is triggered by several stressors, including energy depletion, oxidative stress, and increased temperature [11]. All of these conditions can occur during exercise. However, evidence shows that high‐intensity exercise does not cause markers of eryptosis to increase in well‐trained individuals [12]. On the other hand, enhanced eryptosis has been well described in individuals with SCA [11]. Interestingly, SCT RBCs do not spontaneously become suicidal, but are more susceptible to the eryptotic effects of oxidative stress [13]. Therefore, it is possible that exercise‐induced oxidative stress could trigger eryptosis in individuals with SCT.

Our results also showed that d‐dimer tended to be elevated at baseline, and was significantly elevated at all time points following exercise in the SCT group compared to the controls. These results are in agreement with previous studies that have observed elevated baseline d‐dimer levels in individuals with SCT [14]. On the other hand, a previous study showed that prothrombin time, activated partial thromboplastin time, plasma fibrinogen, and antithrombin III activity were not elevated following a progressive maximal exercise test in SCT compared to control subjects [15]. However, our study used a different, longer exercise protocol, and these are relatively insensitive measures of coagulation activation. Future studies with additional subjects are needed to understand which coagulation parameters are elevated in individuals with SCT compared to controls following exercise.

In conclusion, the results of this exploratory study indicate that vigorous exercise increases MPTF and MP‐prothrombinase activities, suggesting a mechanism through which exercise could increase coagulation activation. Furthermore, exercise resulted in increased RBC PS exposure and elevated numbers of RBCMPs and EMPs in subjects with SCT, which could activate coagulation. A main limitation of the present study was the small number of subjects. Future studies, using larger sample sizes, should be conducted to confirm these findings, and to determine whether increased coagulation activity contributes to microvascular occlusion and an increased risk of exercise‐related complications in SCT.

## CONFLICT OF INTEREST

The authors have declared no conflict of interest.
